# Rapid detection of dermatophytes and *Candida albicans*in onychomycosis specimens by an oligonucleotide array

**DOI:** 10.1186/s12879-014-0581-5

**Published:** 2014-11-07

**Authors:** Huan Wen Han, Mark Ming-Long Hsu, Jong Soo Choi, Chao-Kai Hsu, Hsin Yi Hsieh, Hsin Chieh Li, Hsien Chang Chang, Tsung Chain Chang

**Affiliations:** Institute of Biomedical Engineering, College of Engineering, National Cheng Kung University, Tainan, Taiwan; ESkin Clinic, Tainan, Taiwan; Department of Dermatology, Yeungnam University College of Medicine, Daegu, Republic of Korea; Department of Dermatology, National Cheng Kung University Hospital, Tainan, Taiwan; Department of Medical Laboratory Science and Biotechnology, College of Medicine, National Cheng Kung University, Tainan, Taiwan

**Keywords:** Onychomycosis, Oligonucleotide array, Molecular diagnosis, Internal transcribed spacer, Dermatophytes

## Abstract

**Background:**

Onychomycosis is a fungal infection of nails, leading to the gradual destruction of the nail plate. Treatment of onychomycosis may need long-time oral antifungal therapy that can have potential side effects, thus accurate diagnosis of the disease before treatment is important. Culture for diagnosis of onychomycosis is time-consuming and has high false-negative rates. To expedite the diagnosis, an oligonucleotide array, based on hybridization between immobilized oligonucleotide probes and PCR products, for direct detection of dermatophytes and *Candida albicans* in clinical specimens was evaluated.

**Methods:**

Species-specific oligonucleotide probes designed from the internal transcribed spacer (ITS) regions of the rRNA gene were immobilized on a nylon membrane. The assay procedures consisted of PCR amplification of the ITS using universal primers, followed by hybridization of the digoxigenin-labeled amplicons to probes on the array. Thirty two nail samples (29 patients) were analyzed by the array, and the results were compared with those obtained by culture. Array-positive but culture-negative samples were confirmed by cloning and re-sequencing of the amplified ITS and by reviewing patient’s clinical data. The total recovery of culture and confirmed array-positive but culture-negative results was considered 100% and was used for performance evaluation of both methods.

**Results:**

Concordant results were obtained in 21 samples (10 positives and 11 negatives) by both methods. Eleven samples were array-positive but culture-negative; among them, 9 samples were considered true positives after discrepant analysis. Comparing with culture, the array had significantly higher sensitivity [100% (95% CI 82.2% −100%) *vs* 52.6% (28.9% −75.5%), p <0.001] and negative predictive value [100% (71.3% −100%) *vs* 59.1% (36.4% −79.3%), p <0.05), while no significant differences were observed in specificity (84.6% *vs* 100%, p =0.48) and positive predictive value (90.5% *vs* 100%, p =1.0). The whole procedures of the array were about 24 h, whilst results from culture take 1 to 3 weeks.

**Conclusions:**

The array offers an accurate and rapid alternative to culture. Rapid diagnosis can expedite appropriate antifungal treatment of onychomycosis. However, the single site nature of this study conducted at a referral hospital invites caution.

**Electronic supplementary material:**

The online version of this article (doi:10.1186/s12879-014-0581-5) contains supplementary material, which is available to authorized users.

## Background

Onychomycosis is a fungal infection of nail plate or nail bed. The disease is caused by dermatophytes, nondermatophyte molds, and yeasts, with dermatophytes being the principal pathogens and accounting for 90% of toenail infections [1]. The prevalence of the disease is increasing around the world, presumably due to lifestyle changes and the aging of the population [2]-[6]. It was estimated that more than 10% of the general population had onychomycosis [5]. Toenail onychomycosis is approximately 20 times more common than the disease occurred in fingernail [7], with higher prevalence being found in the elderly [2],[3],[5],[6] and males [7]-[9]. The disease usually does not cure itself and can cause more infectious lesions in other parts of the body.

Although onychomycosis is not life-threatening, it can have adverse effects on patients’ social, emotional, and occupational functioning [3]-[5]. Diagnosis of onychomycosis is routinely performed by detecting fungal elements in nail specimens by microscopy (KOH mount), followed by culture and identification of the infectious fungi [3]. The KOH mount is a rapid diagnostic tool, however the method is nonspecific [3],[10] and can produce considerable false negatives [7],[11],[12]. Culture is the gold standard for diagnosis of onychomycosis, but culture can take 7-15 days or even longer [13] and also has high false-negative rates [11],[14],[15].

Onychomycosis requires long-term antifungal therapies that can result in adverse side effects [3],[11], and hence accurate diagnosis of the disease is important before treatment [11],[13],[16]. Onychomycosis is primarily caused by dermatophytes (mainly *Trichophyton rubrum* and *T. mentagrophytes*) and to the lesser extent by nondermatophyte fungi such as *Acremonium* spp., *Aspergillus* spp., *Fusarium* spp., *Scopulariopsis brevicaulis*, *C. albicans*, *C. parapsilosis*, and other yeasts [3],[6],[17]-[20]*.* Yeasts are more likely to be associated with fingernail infections [2],[21],[22]. The isolation of a dermatophyte is always indicative of infection, but the presence of other molds must be interpreted with care [15].

A variety of molecular methods has been developed to identify the etiological agents of onychomycosis, including PCR-restriction fragment length polymorphism assay [22]-[25], sequencing of specific genes [24],[25], multiplex PCR and real-time PCR [13],[26],[27]. In our previous studies, a wide spectrum of oligonucleotide probes had been designed to identify clinical isolates of molds [28], dermatophytes [29], and yeasts [30]. However, the usefulness of these probes for diagnosis of onychomycosis from direct specimens, rather than pure cultures, is not validated. This study aimed to evaluate the feasibility of a probe-based array to rapidly detect dermatophytes and *C. albicans* from specimens suspected to have onychomycosis.

## Methods

### Clinical specimens

Thirty two nail specimens (28 toenail and 4 fingernail samples) from 29 patients with suspect onychomycosis were analyzed. Samples were collected as part of standard patient care from the Department of Dermatology, National Cheng Kung University Hospital (NCKUH, a tertiary referral hospital), Tainan, Taiwan. The sample collection and test protocols were approved by the Institutional Review Board of NCKUH, with waiver of informed consent. Nail samples were collected as subungual scrapings, clippings, or curettings. Samples were immediately transported in sterile Eppendorf tubes at room temperature to the laboratory of Department of Dermatology, NCKUH, for KOH stain and routine fungal cultures. In addition, 10 toenail samples from 10 healthy persons were used for detection of “background” fungi by our previously constructed mold array [28] and yeast array [30].

### Culture methods

Samples were cultured on Mycosel agar and on Inhibitory Mold agar (both from BBL, Cockeysville, Maryland, USA) at the same day of sample collection [31]. The media were incubated at 25°C for up to 3 to 4 weeks. Species identification was made by their macroscopic and microscopic appearance after staining with lactophenol cotton blue. Direct mounts in KOH were used to determine the presence of yeast and dermatophyte in samples.

### DNA extraction

Each sample was cut into small pieces with a surgical blade. The pieces were transferred to a 2-ml glass homogenizer (Wheaton Science Products, Millville, New Jersey, USA) containing 1 ml of sterilized water. After homogenization, the suspension was transferred to an Eppendorf tube and centrifuged at 8000 × *g* for 10 min. The resulting precipitate was incubated with 0.2 ml of lyticase solution (10 mg/ml; Sigma Aldrich, St. Louis, Minnesota, USA) at 37°C for 30 min. DNA in the lyticase-digested sample was extracted with the Blood & Tissue genomic DNA kit (Viogene, Taipei, Taiwan) according to the manufacturer’s instructions.

### ITS amplification

Amplification of the ITS was performed by a nested PCR. The universal primers V9D (5’-TTAAGTCCCTGCCCTTTGTA-3’) and LS266 (5’-GCATTCCCAAACAACTCGACTC-3’) were used for the first PCR amplification [32]. The PCR reaction mixture (25 μl) consisted 10 mM Tris–HCl (pH 8.8), 50 mM KCl, 0.08% Nonidet P-40 (vol/vol) (Sigma-Aldrich), 1.5 mM MgCl_2_, 1.25 U *Taq* DNA polymerase (Fermentas, Glen Burnie, Maryland, USA), 0.1 mM each deoxyribonucleoside triphosphate, 0.7 μM each primer, and 0.4% (wt/vol) bovine serum albumin (Sigma-Aldrich). The thermocycling condition of PCR consisted of an initial denaturation cycle (94°C, 10 min); 25 cycles of denaturation (95°C, 1 min), annealing (60°C, 1 min), and extension (72°C, 1 min); and a final extension cycle (72°C for 7 min). An aliquot (1 μl) of the PCR reaction mixture was then amplified in the second run of PCR using digoxigenin-labeled primers ITS1 (5’-dig-TCCGTAGGTGAACCTGCGG-3’) and ITS4 (5’-dig-TCCTCCGCTTATTGATATGC-3’) [33]. The first thermocycling condition was followed in the second run of PCR, except that the dTTP concentration was reduced to 0.08 μM and dig-dUTP (0.02 μM, Roche, Mannheim, Germany) was included in the reaction mixture.

### Oligonucleotide probes on the array

**Figure 1:**
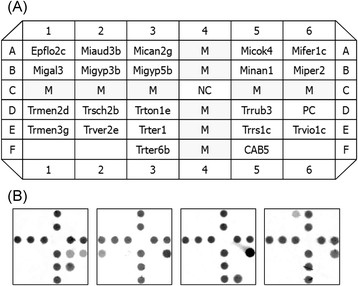
**Layout of oligonucleotide probes on the array and hybridization patterns of 4 samples. A)** Probe codes (species): Epflo2c (*Epidermophyton floccosum*), Miaud3b (*Microsporum audouinii*), Mican2g (*M. canis*), Micok4 (*M. cookei*), Mifer1c (*M. ferrugineum*), Migal3 (*M. gallinae*), Migyp3b and Migyp5b (*M. gypseum*), Minan1 (*M. nanum*), Miper2 (*M. persicolor*), Trmen2d and Trmen3g (*Trichophyton mentagrophytes*), Trsch2b (*T. schoenleinii*), Trton1e (*T. tonsurans*), Trrub3 and Trrs1c (*T. rubrum*), Trver (*T. verrucossum*), Trter1 and Trter6 (*T. terrestre*), Trrs1c (*T. soudanense*), Trvio1c (*T. violaceum*), CAB5 (*Candida albicans*), PC (positive control), NC (negative control), and M (marker). **B)** Hybridization patterns of 4 positive samples from patients with onychomycosis. From left to right, *Trichophyton rubrum*, *T. mentagrophytes*, *C. albicans*, and *Microsporum canis*.

A total of 22 oligonucleotide probes were spotted on nylon membrane to form an array (0.6 cm by 0.6 cm). Among the 22 probes, 20 were used to identify 17 species of dermatophytes [29], one was used to identify *C. albicans*[30], and the remaining one was fungus-specific [29]. Oligonucleotide probes were diluted 1:1 with a tracking dye solution to a final concentration of 10 μM, spotted onto a positively charged nylon membrane (Roche, Mannheim, Germany) by an arrayer (Ezspot SR-A300; EZlife Technology, Taipei, Taiwan) with a solid pin (400 μm in diameter). Once all probes had been applied, the membrane was air-dried and exposed to shortwave UV (Stratalinker 1800; Stratagen, La Jolla, California, USA) for 30 s to fix the probes on the membrane [28]. The layout of probes on the array is shown in Figure 1(A).

### Array hybridization

The procedures for prehybridization, hybridization (55°C for 90 min), and colorimetric detection with alkaline phosphatase-conjugated anti-digoxigenin antibodies were described previously [28]. The hybridized spots with a diameter of 400 μm could be read by the naked eye. A sample was considered positive for a dermatophyte (or *C. albicans*) when the species-specific probe (or probes) and the positive control probe (fungus-specific) were simultaneously hybridized. To detect “background” fungi, the DNAs in nail samples from healthy persons were extracted, amplified, and hybridized with a mold array [28] and a yeast array [30] in a similar way.

### Discrepant analysis

If a sample was positive by the array but negative by culture, cloning and re-sequencing of the amplified ITS were performed to resolve the discrepancy. The ITS was amplified by a nested PCR according to the conditions described in the previous section except that primers ITS1 and ITS4 used in the second run of PCR were not labeled with a digoxigenin molecule at their 5’ ends. The amplicon was cloned with the pOSI-T PCR Cloning Kit (GMbiolab, Taipei, Taiwan) following the manufacturer’s instructions. For each discrepant sample, the ITS was re-amplified from 30 positive clones by PCR using primers ITS1 and ITS4 [32] and sequenced on a 3130*xl* genetic analyzer (Applied Biosystems, Taipei, Taiwan). The resulting sequences were used for searching homologous sequences in public databases for species identification. If the cloning experiment failed to confirm the result of an array-positive but culture-negative sample, the following clinical findings, if applicable, were used to solve a discrepancy: the result of KOH mount, the contraction of tinea pedis in the patient, and the patient’s outcome after antifungal treatment. An array-positive but culture-negative sample was regarded as a true positive if the ITS cloning experiment was positive, or at least two of the three clinical findings (KOH mount, presence of tinea pedis, and improvement of onychomycosis after treatment) were positive.

### Performance calculation and statistics

The total recovery of dermatophytes and *C. albicans* from culture and from confirmed positive results of the array was considered 100% and used for performance evaluation of the array and culture methods. Sensitivity, specificity, positive (PPV) and negative predictive value (NPV) were calculated after discrepant analysis. Significant difference was estimated by Fisher's exact test.

## Results

### Fungi detected in healthy toenails

Of the 10 toenail samples from healthy persons, no dermatophyte or *C. albicans* was found in any sample by the array. However, a wide spectrum of nondermatophyte molds (*Aureobasidium pullulans*, *Aspergillus versicolor*, and *Exophiala werneckii*) and yeasts (*Candida rugosa*, *C. parapsilosis*, *Cryptococcus albidus*, and *Rhodotorula rubra*) were detected by the mold and yeast arrays previously established in our laboratory [28],[30]. These microorganisms could be considered “background” fungi or normal flora of healthy toenails.

### Detection of dermatophytes and *C. albicans*in clinical samples

Thirty two samples (29 patients) with suspect onychomycosis were parallel analyzed by the array and culture. Concordant results were found in 21 samples (10 positives and 11 negatives) by both methods (Table 1). Of the 10 concordant positive samples, *T. rubrum*, *T. mentagrophytes*, *Microsporum canis*, and *C. albicans* were detected in 5, 1, 1, and 3 samples, respectively, and their hybridization patterns are shown in Figure 1(B). Eleven samples were only positive by the array, with *T. rubrum* being detected in 10 samples and *C. albicans* in one sample (C36) (Table 2). The ITS cloning experiment confirmed the presence of *T. rubrum* in 7 (C26, C27, C29, C30, C32, C38, and C50) of the 11 array-positive but culture-negative samples (Table 2). However, the cloned sequences of the remaining 4 samples (C28, C36, C46, and C49) did not match species (dermatophytes or *C. albicans*) detected by the array.

**Table 1:** **Comparison of the array and culture for diagnosis of onychomycosis**

Method	No. of specimens with culture result		No. of specimens after discrepant analysis	
	Positive	Negative	Positive	Negative
**Array**				
Positive	10	11	19	2
Negative	0	11	0	11
**Culture**				
Positive			10	0
Negative			9	13

**Table 2:** **Discrepant analyses of 11 samples that were array-positive but culture-negative**

Sample	Fungus detected by		ITS cloning (%)^a^	KOH mount/tinea pedis^b^	Improvement after treatment	Final interpretation of the array result
	Culture	Array				
C26	*Aspergillus* sp.	*Trichophyton rubrum*	*T. rubrum* (99.8)	ND/+^c^	(follw-up loss)	True positive
C27	(negative)	*T. rubrum*	*T. rubrum* (99.2)	ND/+	Yes	True positive
C28	*Penicillium* sp.	*T. rubrum*	(negative)	-/-	(follw-up loss)	False positive
C29	*Cladosporium* sp.	*T. rubrum*	*T. rubrum* (100)	ND/+	Yes	True positive
C30	(negative)	*T. rubrum*	*T. rubrum* (100)	ND/-	(follw-up loss)	True positive
C32	*Candida* sp*.*	*T. rubrum*	*T. rubrum* (99.6)	+/-	Yes	True positive
C36	(negative)	*C. albicans*	(negative)	-/-	Yes^c^	False positive
C38	(negative)	*T. rubrum*	*T. rubrum* (100)	+/+	Yes	True positive
C46	(negative)	*T. rubrum*	(negative)	+/+	(follw-up loss)	True positive
C49	(negative)	*T. rubrum*	(negative)	+/+	Yes	True positive
C50	(negative)	*T. rubrum*	*T. rubrum* (100)	+/+	Yes	True positive

^a^Values in parentheses are percentages of sequence similarities of the determined sequences with the best-scoring sequences in public database.

^b^ND, not determined. +, KOH mount positive or the corresponding patient was positive for tinea pedis. -, KOH mount negative or the corresponding patient was negative for tinea pedis.

^c^The patient was not treated with antifungals, but his onychomycosis completely resolved in the follow-up visit.

Although negative by the ITS cloning experiment, samples C46 and C49 were KOH mount positive and were from patients with tinea pedis; therefore the two samples were considered true positives by the array according to definition. The onychomycosis of one patient (sample C49) improved after antifungal treatment, but another patient (sample C46) was lost during follw-up (Table 2). The detection of *T. rubrum* in sample C28 by the array was a false positive, since all confirmation results (ITS cloning, KOH mount, and tinea pedis) were all negative and the patient was lost during follw-up (Table 2). The detection of *C. albicans* in sample C36 by the array was also a false positive, because the results of ITS cloning, KOH mount, and tinea pedis were all negative in this sample. No antifungal was prescribed for this patient (sample C36), but the patient’s onychomycosis completely resolved at the follow-up visit. In summary, except 2 samples (C28 and C36), 9 of the 11 array-positive but culture-negative samples were true positives after discrepant analyses.

### Performance of the array

After resolving discrepancies, the array had 19 true positives and 2 false positives (Table 1). The sensitivity, specificity, PPV, and NPV of the array were 100%, 84.6%, 90.5%, and 100%, respectively, while the corresponding values of culture were respectively 52.6%, 100%, 100%, and 59.1% (Table 3). The sensitivity (p <0.01) and NPV (p <0.05) of the array were significantly higher than those of culture, however no significant differences in specificity and PPV were observed between the two methods.

**Table 3:** **Performance of the array and culture for diagnosis of onychomycosi**s

Method	Performance (95% confidence interval), %			
	Sensitivity	Specificity	Positive predictive value	Negative predictive value
**Array**	100 (82.2-100)**	84.6 (54.5-97.6)	90.5 (69.6-98.6)	100 (71.3-100)*
**Culture**	52.6 (28.9-75.5)	100 (75.1-100)	100 (69.0-100)	59.1(36.4-79.3)

**p <0.01 by 2-tailed Fisher exact test.

*p <0.05 by 2-tailed Fisher exact test.

## Discussion

The feasibility of using an array for rapid detection of dermatophytes and *C. albicans* in clinical specimens with suspect onychomycosis was evaluated and satisfactory results were obtained. The current method, with a much shorter turnaround time, offers significant advantages of speed and sensitivity over conventional culture method. It was estimated that a medical technician can easily handle 20 specimens during a time frame of 24 h.

As found in this study (Table 1) and in previous reports [8],[34], a wide spectrum of commensal or transiently colonizing fungi can be found from healthy nails. These commensal fungi have a potential to overgrow dermatophytes or other real pathogens during culture, even if antibiotics and antifungals are included in selective media [31]. In this study, 4 of the 11 array-positive but culture-negative samples were contaminated (growth of bacteria or nondermatophytes) during culture (data not shown). The current array seems to have a better ability to detect fungal pathogens in a complex flora; this advantage was also demonstrated in a study using an array to detect fungal pathogens in patients with cystic fibrosis [32].

In this study, *Microsporum canis* was detected in a sample (data not shown) from an immunocompromised female aged 36. The patient also contracted tinea pedis and tinea corporis. *M. canis* predominantly infects the human hair, scalp, and trunk, but rarely infect nails [35],[36]. The infection of *M. canis* was presumably due to the altered immune status of the patient [37], who well responded to antifungal treatment (terbinafine) during a course of 7 weeks. *Epidermophyton floccosum* was not found in this study, although the microorganism was a common etiological agent of onychomycosis in boarding school residents [38].

Two array-positive but culture-negative samples (C46 and C49) were considered true positives, based on the findings of positive KOH preparations and the contraction of tinea pedis in the two patients. Tinea pedis has a high correlation with onychomycosis [5],[39],[40]; the disease can be used as a predictive risk factor for contracting onychomycosis. The secondary spread of fungi from the infected foot may lead to the infection of heel, nail plates, sole, toes, and web spaces [39]. Histopathological examination of the nail plate has been demonstrated as a useful complementary technique for diagnosing onychomycosis [11],[41], especially when there is a strong clinical suspicion but fungal culture and KOH mount are negative. However, the technique was not a routine clinical practice when the study was conducted in our hospital; therefore the results of histopathological examination were not available for discrepant analysis or used as a gold standard for method comparison. But, very recently, the histopathological examination technique of dermatological specimens (41) had been gradually adopted in the Department of Dermatology in our hospital.

The limitation of the current study was the relatively small sample size (32) used, partially due to the labor-intensive work of the ITS cloning experiment when discrepant results occurred between methods. In addition, as this study was conducted at a tertiary medical center, further studies of the array are needed before the method is implemented in primary or secondary hospitals or resource-poor settings for diagnosis of onychomycosis.

## Conclusions

As identification of the fungal species is not routinely performed before pretreatment of onychomycosis, the array can be valuable (easy, rapid, cheap and accurate) in clinical use. The current method, having a good sensitivity and a short turnaround time, can expedite the diagnosis of onychomycosis and thus has a potential to improve the outcome of onychomycosis.

## Authors’ contributions

MMH and TCC designed the study protocol. CKH, MMH, HCC, JSC, and TCC decided the strategies for specimen collection and diagnosis. HCL, HYH, and HWH performed and optimized the experimental procedures of array hybridization. HWH did the discrepant analyses. HWH and TCC analyzed the results and wrote the manuscript. All authors read and approved the final manuscript.

## Authors’ original submitted files for images

Below are the links to the authors’ original submitted files for images. Authors’ original file for figure 1

## Abbreviations

Dig: Digoxigenin
ITS: Internal transcribed spacer
NPV: Negative predictive value
PPV: Positive predictive value
