# Supplementary figures and images for: Rapid detection of dermatophytes and Candida albicansin onychomycosis specimens by an oligonucleotide array
Source: BMC Infect Dis. 2014 Nov 7;14:581. doi: 10.1186/s12879-014-0581-5 (PMC4234842; doi:10.1186/s12879-014-0581-5)

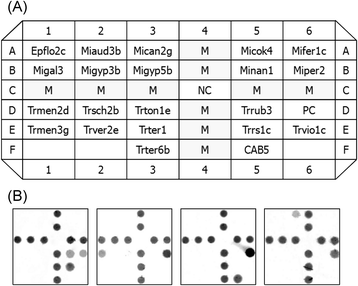

Supplement: Supplementary file 1 — Authors’ original file for figure 1 [file 12879_2014_581_MOESM1_ESM.gif]
